# Resuscitation by hyperbaric exposure from a venous gas emboli following laparoscopic surgery

**DOI:** 10.1186/1757-7241-20-51

**Published:** 2012-08-03

**Authors:** Thomas Kjeld, Egon G Hansen, Nana G Holler, Henrik Rottensten, Ole Hyldegaard, Eric C Jansen

**Affiliations:** 1 Department of Cardiology, University of Copenhagen, Herlev Hospital, Herlev Ringvej, 100 DK-2730, Denmark; 2 Department of Anaesthesiology and intensive care, University of Copenhagen, Herlev Hospital, Herlev Ringvej, 100 DK-2730, Denmark; 3 The Hyperbaric Unit, Department of Anaesthesiology, Centre of Head and Orthopaedics, Rigshospitalet, University of Copenhagen, Blegdamsvej 9, 2100, Copenhagen Ø, Denmark

**Keywords:** CO_2_ embolism, Cardiac arrest, Hyperbaric oxygen treatment, Laparoscopy

## Abstract

Venous gas embolism is common after laparoscopic surgery but is only rarely of clinical relevance. We present a 52 year old woman undergoing laparoscopic treatment for liver cysts, who also underwent cholecystectomy. She was successfully extubated. However, after a few minutes she developed cardiac arrest due to a venous carbon dioxide (CO_2_) embolism as identified by transthoracic echocardiography and aspiration of approximately 7 ml of gas from a central venous catheter. She was resuscitated and subsequently treated with hyperbaric oxygen to reduce the size of remaining gas bubbles. Subsequently the patient developed one more episode of cardiac arrest but still made a full recovery. The courses of events indicate that bubbles had persisted in the circulation for a prolonged period. We speculate whether insufficient CO_2_ flushing of the laparoscopic tubing, causing air to enter the peritoneal cavity, could have contributed to the formation of the intravascular gas emboli. We conclude that persistent resuscitation followed by hyperbaric oxygen treatment after venous gas emboli contributed to the elimination of intravascular bubbles and the favourable outcome for the patient.

## Introduction

Laparoscopic surgery with carbon dioxide (CO_2_) insufflation affects circulatory, pulmonary, renal, splanchnic, and endocrine functions but such perturbations are, most often, of little clinical significance [1]. Yet cardiac arrest has been reported following 2–20 of 100,000 laparoscopic procedures [2], and cardiac arrest seems to be related to a vasovagal response following rapid peritoneal distension and gas embolism [3]. Human and animal studies of laparoscopic surgery indicate gas embolism in about half of the cases, as detected by transoesophageal echocardiography [4-6]. Symptomatic supportive care is the primary therapeutic modality for venous gas embolism (VGE), while hyperbaric oxygen therapy is the first line of treatment for arterial gas embolism [7,8]. Here, we report of a patient who survived significant gas embolism by aspiration of the embolus followed by hyperbaric oxygen treatment.

## Case report

A female patient (52 years, 56 kg, ASA II) underwent laparoscopic marsupialization for liver cysts and also cholecystectomy. The patient was undergoing treatment with thiazide diuretics and a renin-angiotensin inhibitor for hypertension but had no other illnesses as evaluated by routine laboratory tests. The patient was pre-medicated with oral dexamethasone 4 mg. Anaesthesia was induced with propofol 200 mg and maintained with propofol and remifentanil. After induction of anaesthesia, oral intubation was facilitated with rocuronium 40 mg, which was increased to a total dose of 90 mg guided by ‘train of four’ [9]. The ventilation was controlled with an inspired oxygen fraction (FiO_2_) of 0.50, end-tidal tension of carbon dioxide (*ET’*_CO2_) was between 28 and 34 mmHg and a positive end-expiratory pressure (PEEP) of 5 cm H_2_O. Blood pressure was monitored noninvasively on the right arm and oxygen saturation was measured by a pulse oximeter.

**Figure 1:**
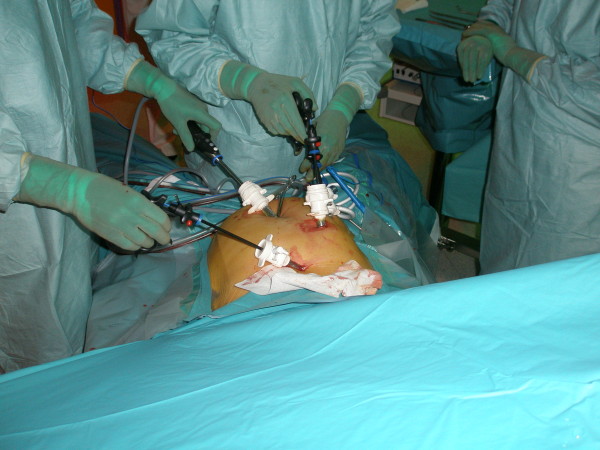
**Laparoscopic procedure.** Trocars are inserted in the abdomen (not from the presented case, picture kindly supplied by Dr. E. G. Hansen).

**Figure 2:**
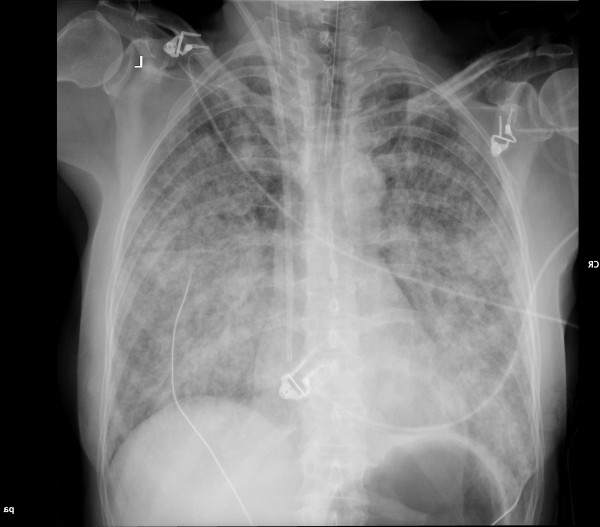
X-ray of the chest of the patient in the presented case showing bilaterally pulmonary oedema.

Four trocars were inserted in the abdomen and one was connected to a CO_2_ source (Fig. 1). Intra-abdominal pressure was maintained < 12 mmHg with the patient in a head-up tilted position. During dissection, the surgeon used an ABC surgical system (Force Argon™ II; Valleylab™, Boulder, CO). When awake after the operation, the patient complained of nausea. After a few minutes, the patient developed ventricular tachycardia and then pulseless electrical activity (PEA). Advanced resuscitation included uninterrupted manual compression, immediate intravenous administration of 1 mg of adrenaline and 3 mg of atropine intravenously, followed by 1 mg of adrenaline every 2–4 min. The patient was reintubated and received 100% oxygen. Transthoracic echocardiography showed minimal contraction of the ventricular walls. Gas bubbles were seen to move from the inferior caval vein into the right atrium and ventricle. The patient was placed in Trendelenburg’s position and a central venous catheter was placed through the internal jugular vein. Approximately 7 ml of gas was aspirated through the catheter. Resuscitation was successful after 40 min and she was transferred to the intensive care unit (ICU) with assisted mechanical ventilation: PEEP 13 cm H_2_O and a FiO_2_ of 1.0. Frothy pink sputum was seen in the tracheal tube and a chest X-ray showed bilaterally pulmonary oedema (Fig. 2). Blood pressure was measured by a radial arterial catheter and maintained at 90/50 mmHg by intravenous infusion of noradrenaline 25–50 μg/kg/h.

The patient was referred to a nearby hospital and treated in a hyperbaric chamber at a pressure of 2.8 ATA. The patient was ventilated with 100% oxygen. Within 2 h and 37 min, the fluid balance was 1000 ml positive and the inotropic medication was discontinued when systolic pressure was 140 mmHg. The patient was sedated with fentanyl 2 ml/h and propofol 20 ml/h [10] and by then no longer had frothy pink sputum. When the pressure was lowered to 1.9 ATA the patient developed non-sustained ventricular fibrillation followed by bradycardia.

It was believed that the ventricular fibrillation and subsequent cardiac arrest was a result of reperfusion rather than VGE and the hyperbaric treatment was therefore terminated.

The patient was referred to the ICU where, on arrival, she again developed cardiac arrest with PEA. Treatment was with adrenaline 1 mg, atropine 3 mg, and uninterrupted manual compression while mechanical ventilation with 100% oxygen was continued. Transthoracic echocardiography showed no movement of the ventricular walls and extracorporeal membrane oxygenation was considered, while adrenaline administration was repeated every 2–4 min. The patient then went into ventricular fibrillation, but after one biphasic direct current shock of 200 J, return of cardiac function was obtained after 18 min. The patient was then monitored according to a hypothermia algorithm that included continuous ECG, a central venous catheter and an arterial catheter. Temperature was monitored using a Foley catheter. Goals of treatment were a mean arterial pressure (MAP) of 65–100 mmHg (by continuous dopamine infusion, 2–10 μg/kg/min), heart rate 40–90 min − ^1^, central venous pressure 2–10 mmHg, blood glucose 4.5–6.1 mmol/L, and diuresis > 1 mL/kg/h. Active cooling was initiated with ice packs and infusion of 30 mL/kg of 4°C Ringer’s solution as well as continued therapeutic surface cooling using an Allon Thermowrap ^TM^ (MTRE, Israel). The target core temperature was 32.5–35.5°C and it was maintained for 24 h with subsequent rewarming by 0.5°C per hour until normothermia. Meanwhile, the patient was sedated to a Ramsey score 6 (propofol 0.3–4.0 mg/kg/h, fentanyl 100 μg/h) and paralysis during muscle shivering (cisatracurium, 0.06–0.12 mg/kg/h) [11].

On post-operative day (POD) 1, arterial blood gas analysis and chest X-ray findings were normal. On POD 2, the patient was extubated and transferred to a cardiology unit and she made a full recovery on POD 4. Repeated transthoracic echocardiography showed a normal ejection fraction, no valvulopathy, pulmonary hypertension, or pericardial exudations, but a possible small ventricular septum defect. ECG showed no sign of infarction. Ultrasonography of the liver revealed no sequelae and clinical neurological assessment was normal.

## Discussion

The risk of iatrogenic gas embolism is low. Three cases per 100,000 may be in need of hyperbaric treatment for gas embolisms and only 7/4,727,496 cases are described as being caused by laparoscopic surgery with intraperitoneal insufflation of CO_2_[8].

However, in the case presented here, the patient went through laparoscopic liver surgery and cholecystectomy with an inherent risk of VGE, since liver veins are little prone to collapse in the supine position [12,13]. The effect of VGE depends on the rate of CO_2_ infusion and its volume [14]. For adults, the potentially lethal volume is estimated at 200–300 ml or 3–5 mL/kg [15]. The effect VGE also depends on whether the patient is breathing spontaneously, yielding a negative thoracic pressure during inspiration and hence facilitating air entrapment, or whether the patient is under positive pressure ventilation, possibly supplemented by PEEP.

The essential pathological factor is pulmonary gas embolism. Tachyarrhythmias are common following VGE and the electrocardiogram demonstrates a right heart strain pattern as well as ST–T changes, and blood pressure decreases with cardiac output. Pulmonary symptoms following VGE include dyspnoea, continuous coughing, and chest pain. Gasping for air reduces the intrathoracic pressure and may be a sign of air entrapment. Pulmonary signs of VGE include rales, wheezing, and tachypnoea. The central nervous system may be affected by VGE by cardiovascular collapse secondary to reduced cardiac output from right ventricular failure or myocardial ischemia, which rapidly results in cerebral hypoperfusion [16]. In the present case, the VGE did probably not pass the interventricular septum, as no gas was seen on the left side of the heart and the patient recovered without any apparent neurological deficits despite three episodes of cardiac arrest and prolonged resuscitation.

The most widely used gas for laparoscopic procedures is CO_2_[2]. CO_2_ is inexpensive, non-flammable and has the additional advantage of being readily available. Since CO_2_ has a high Ostwald’s solubility coefficient in blood and tissue (L = 0.54 ml per ml whole blood [17]), and since blood holds a high buffering capacity for CO_2,_ VGE’s consisting of CO_2_ are quickly absorbed should they occur. When insufflated into the abdominal cavity, CO_2_ diffuses across the peritoneum, and, more importantly, is carried by the circulation to the lungs and expired. However, as demonstrated by SP Taylor and GM Hoffman, substantial amounts of air (containing 79% of the insoluble N_2_[17]) may be insufflated into the peritoneal cavity from the tubing, if the system is not adequately purged with CO_2_ gas before insertion [18,19]. The VGE in the present case may therefore be a combination of a CO_2_ and air embolism. Whether or not air contamination of the peritoneal cavity was a matter of significance in the present case remains speculative, as we were not capable of measuring the gas content of the bubbles aspirated through the central venous catheter. Although surgeons routinely purge laparoscopic tubing systems with CO_2_ gas, as was the standard operational procedure in the present case, surgeons and anaesthetists should be aware of the risk of simultaneous CO_2_ and air embolisms during these procedures.

In the case of air bubble contamination during laparoscopic procedures, AB Branger et al. [16] have demonstrated that microscopic N_2_ bubbles (50 nl) having a cylindrical shape in the capillary vessels will dissolve in > 300 min. Larger emboli, in the size of millilitres, would require even longer resorption time if left untreated, and air bubbles have been observed to persist within the vasculature for more than 48 h [20]. The molar flux of any permeating ideal gas species present in blood and tissues obeys Fick’s first law of diffusion [21,22] whereby each gas will have a flux through the surface area of the gas emboli, driven by the partial pressure difference between the inside of the emboli and surrounding tissue and blood. It is conceivable that larger gas emboli of CO_2_ may persist in blood vessels and tissues long enough for them to equilibrate with other gases present in blood and tissues (i.e. oxygen, nitrogen and water vapour), thereby increasing the bubble resolution time considerably, as demonstrated by Barrera et al. [23]. During anaerobic metabolism, such as with gas embolism blocking microcirculation, large amounts of CO_2_ would be liberated from bicarbonate stores of blood and tissue, resulting in high levels of local PCO_2_, thereby reducing the partial pressure gradient of CO_2_ from the emboli to blood, which could contribute considerably to the bubble volume or reduce the rate of CO_2_ disappearance from the emboli [24].

If large iatrogenic VGE causes hemodynamic changes, treatment in a hyperbaric chamber combined with 100% oxygen breathing is well indicated [14,25,26]. If available, such treatment will often consist of compression of the patient to 285 kPa (i.e. 2.8 atm abs) breathing oxygen. The purpose is to *1*) reduce the intravascular bubble size by compression of the gas phase according to Boyles Law, *2*) maximize gas partial pressure gradients from bubbles and tissues to blood thereby increasing the speed of bubble elimination, and *3*) to introduce systemic hyperoxia in an otherwise hypoxic tissue. The first goal is almost immediately achieved by compression regardless of the breathing mixture used, whereas oxygen breathing would achieve the latter two goals. An arterial partial pressure of oxygen greater than 2000 mmHg is readily achieved. Hyperoxia also increases the distance of oxygen diffusion in tissues and offsets the embolic insult to the microvasculature. Hyperbaric oxygen is the treatment of choice for arterial gas embolism [14]. Time is important, but delayed treatment in a hyperbaric chamber may still be indicated to ameliorate the patient’s condition and reduce long term neurological sequelae.

Apparently, the patient benefited from the hyperbaric treatment. The courses of events suggest that bubbles had persisted in the circulation for a prolonged period, and that persistent resuscitation followed by hyperbaric treatment after VGE contributed to their elimination. The second cardiac arrest might have been avoided, if the pressure in the chamber had been increased instead of reducing the pressure to 1.9 ATA.

## Consent

The patient has given verbal and written consent to the publication of her case report.

## Competing interests

The author(s) declare that they have no competing interests.

## Authors’ contributions

OH contributed to the drafting of the manuscript including interpretation of observed clinical data as well as search of and adding of references. TJ contributed in writing and editing of manuscript, search of and adding references, correspondance with co-authors and adjustments according to comments from editors from SJTREM and biomed central, adding figure 2 and observed clinical data. ECJ contributed to the drafting of the manuscript including interpretation of observed clinical data as well as search of and adding of references. HR contributed to the drafting of the manuscript including interpretation of observed clinical data. NGH contributed to the interpretation of observed clinical data. All authors read and approved the final manuscript.
